# Keraring Intrastromal Segment Depth Measured by Spectral-Domain Optical Coherence Tomography in Eyes with Keratoconus

**DOI:** 10.1155/2017/4313784

**Published:** 2017-02-02

**Authors:** Ugo de Sanctis, Carlo Lavia, Marco Nassisi, Savino D'Amelio

**Affiliations:** ^1^Department of Surgical Sciences, Eye Clinic, University of Turin, Turin, Italy; ^2^Department of Eye Diseases, Ophthalmic Hospital of Turin, Turin, Italy

## Abstract

*Purpose*. To evaluate agreement between measured and intended distance of Keraring (Mediphacos, Belo Horizonte, Brazil) intracorneal ring segments from the anterior and posterior corneal surfaces.* Methods*. Twenty-six Keraring ICRS implanted in 24 keratoconic eyes were examined. The distance from the Keraring apex to the anterior corneal surface and the distance from the inner and the outer corners to the posterior corneal surface were measured 3 months postoperatively using spectral-domain optical coherence tomography. Agreement between measured distance and intended distance was assessed by calculating the absolute differences and 95% limits of agreement (95% LoA).* Results*. The mean absolute difference was significantly lower (*p* < 0.001) for the measurements taken at the inner corner (23.54 ± 15.90 *μ*m) than that for those taken at the apex (108.92 ± 62.72 *μ*m) and the outer corner (108.35 ± 56.99 *μ*m). The measurements taken at the inner corner were within ±25 and ±50 *μ*m of the intended distance in 15/26 (57.7%) and 24/26 (92.3%) cases, respectively, and showed the narrowest 95% LoA with the intended distance (−57.61 to 55.15 *μ*m).* Conclusions*. The distance of the inner corner from the posterior corneal surface showed the best agreement with the intended distance. This measurement is suitable for determining whether the actual Keraring depth matches the intended depth.

## 1. Introduction

Intrastromal corneal ring segments (ICRS) are used for the surgical treatment of corneal ectasia [1–3]. These space-occupying elements are implanted into a tunnel created in the deep stroma at the midperiphery of the cornea. Segment implantation generates an arc-shortening effect that flattens the central cornea and reduces curvature asymmetry [4–6]. The depth of ICRS placement is planned according to individual corneal thickness and is crucial for achieving efficacy and safety of the procedure. Incorrect depth results in unpredictable changes in corneal curvature and increases the risk of complications such as corneal perforation, superficial erosion, and ring extrusion [7, 8].

The depth of ICRS in the corneal stroma can be assessed with different methods, including slit lamp biomicroscopy, rotating Scheimpflug camera, and optical coherence tomography (OCT). Slit lamp biomicroscopy is based on examiner impression and does not allow precise and quantitative estimation of the implant depth [8]. OCT examination permits imaging of the cornea at a higher resolution than the rotating Scheimpflug camera and measurement of the ICRS distance from the anterior and the posterior corneal surface.

A widely used implant is the Keraring (Mediphacos, Belo Horizonte, Brazil) ICRS, which is made of polymethyl methacrylate (PMMA) and has a triangular cross-section [2, 3]. It is inserted with the apex facing the anterior corneal surface and the base facing the posterior corneal surface. Several authors used the OCT to measure the distance from Keraring to the anterior corneal surface [9–11]. To the best of our knowledge, this is the first study to investigate the implant distance also with reference to the posterior corneal surface. We then evaluated the agreement between the measured and the intended distance to determine which measurement might be suitable to verify that segment depth matches intended depth.

## 2. Patients and Methods

This prospective observational case series comprised 24 eyes of 21 consecutive patients (mean age 35.2 ± 8.4 years, range 22–54) with keratoconus that underwent Keraring ICRS implantation at the Eye Clinic of Turin University between July 2014 and September 2015. The study was approved by the Institutional Review Board of the Ophthalmic Hospital of Turin and followed the tenets of the Declaration of Helsinki. The patients signed a consent form and they were aware of the nature of the study.

The patients were recruited at the Cornea Service according to the following criteria: age > 18 years, dissatisfaction with spectacle-corrected vision, and intolerance to contact lens use. Patients with corneal thickness < 400 *μ*m, central corneal scarring, presence of other corneal diseases, and previous ocular surgery were excluded.

Preoperative data, surgical details, and postoperative results were recorded on a standardized form and entered in a computerized database. Preoperative data included patient age, Snellen uncorrected and corrected distance visual acuity (UDVA and CDVA), mean keratometry (Km) measured on a ring of 15° around the corneal apex (Pentacam HR, Oculus, Germany), and corneal thinnest point in the hypothetical tunnel area as measured by means of the OCT pachymetry map (RTVue 100, Optovue, USA). Surgical details included Keraring characteristics (model, arc length, and thickness), incision depth for tunnel creation, and intraoperative complications. Postoperative data included Snellen UDVA and CDVA, Km, and any complications.

### 2.1. Surgery

The surgical procedure was carried out under sterile conditions and topical anesthesia by a single surgeon (U.d.S.). The intrastromal tunnel was created via manual technique in 19 eyes and femtosecond laser technology (IntraLase 150 kHz, Advanced Medical Optics, Santa Ana, CA, USA) in 5 eyes. Preoperatively the corneal vertex was marked with a methylene blue-tinted Sinskey hook. In the manual technique, the tunnel was outlined on the corneal surface with a circular marker. A 1-mm entry incision was made with a calibrated, diamond square-bladed knife on the steepest axis. The incision depth was set at 80% of the thinnest point measured in the tunnel area. The corneal tunnel was then created with curved corneal dissectors. For the femtosecond laser procedures, the energy was set at 1.5 mJ to create a 1.4-mm entry incision on the steepest axis and the stromal tunnel. The incision and the tunnel depth were set at 70% of the thinnest point measured in the tunnel area. The Keraring ICRS were implanted with the manufacturer's forceps. The segment arc length and thickness were selected according to the manufacturer nomograms (other cited materials: Keraring Calculation Guidelines 2009; http://smmedical.cl/wp-content/uploads/2013/10/Agrupado.pdf). At completion of surgery, a bandage contact lens was placed on the cornea. Tobramycin 0.3% and dexamethasone 0.1% eye drops were prescribed 3 times a day for 1 month. Postoperative visits were scheduled at 1, 7, 30, and 90 days after surgery.

### 2.2. Spectral-Domain OCT Corneal Examination

One investigator (M.N.) scanned each cornea with RTVue100 (software version 2.6). This spectral-domain OCT uses a superluminescent diode (*ƛ* 830 nm) as light source. The effective acquisition speed is 26.000 A-scans/s. The depth and the transverse resolution are 5 *μ*m and 15 *μ*m, respectively. The instrument was equipped with the low magnification lens of the Corneal Adaptor Module that provides a scan length of 6 mm and a scan depth of 1.96-mm.

The Corneal Adaptor Module allows corneal examination with different scan patterns. The pachymetry map pattern was used to measure the thinnest point in the hypothetical tunnel area a few days before surgery. The high definition (HD) line pattern was used for measuring the Keraring depth inside the corneal stroma at 3 months after implantation. Using these scan patterns, the instrument software transforms the OCT optical images into physical images of the cornea by means of a dewarp calculation that takes into account corneal curvature and the index of refraction of the media [12, 13]. Only good quality scans showing a signal strength index (>30) were accepted as valid.

**Figure 1:**
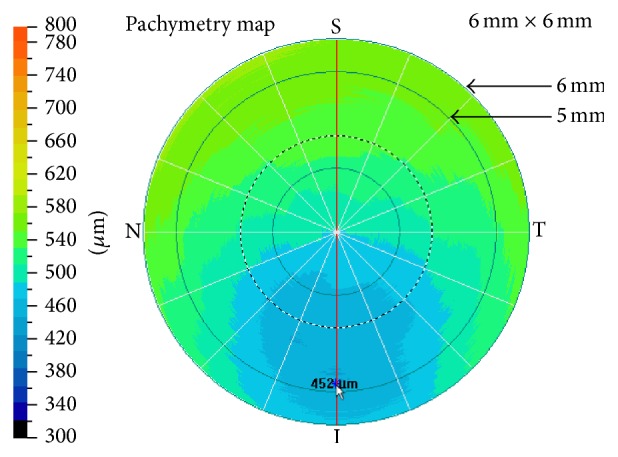
The pachymetry map in RTVue 100 is divided into a central circular area (0–2 mm), sixteen pericentral sectors (2–5 mm), and sixteen transitional sectors (5-6 mm). To find the thinnest point in the hypothetical tunnel area, the mouse cursor was slowly dragged along the transitional sectors between the 5-mm and the 6-mm ring. The lowest value found in that area was recorded as the thinnest point (452 *μ*m in this case).

The pachymetry map pattern includes 8 meridional cross-section scans (6 mm in length, 1024 A-scans each) at 22.5-degree intervals automatically captured in 0.32 seconds. The computer algorithm generates a corneal thickness profile from each meridional scan and computes a color-coded pachymetry map by interpolation. The pachymetry map was centered on the corneal vertex and displayed on the instrument monitor. The examiner then slowly dragged the mouse cursor along the hypothetical tunnel area to find the thinnest point and calculate the incision depth (Figure 1).

**Figure 2:**
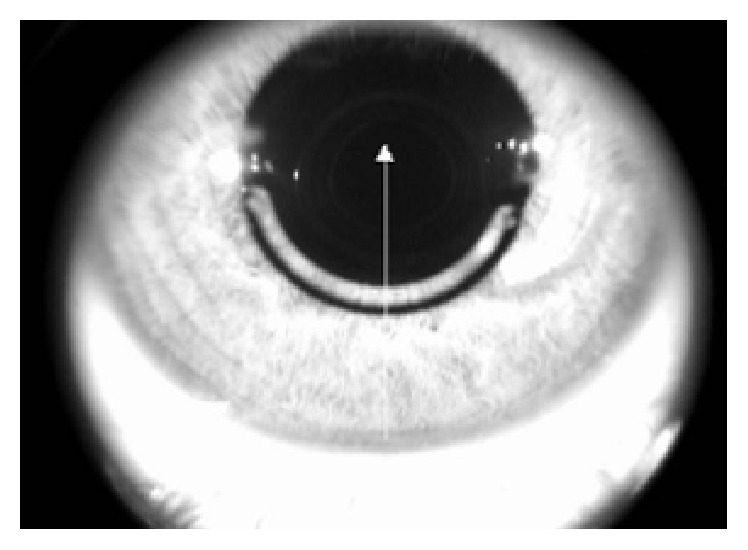
Enface OCT image of the Keraring showing the HD line scan aligned on the segment center and corneal vertex.

The HD line pattern includes one meridional cross-section scan of the cornea (6 mm in length, 4096 A-scans) acquired automatically in 0.16 seconds. The scan was aligned so that it passed through the corneal vertex and Keraring center (Figure 2).

**Figure 3:**
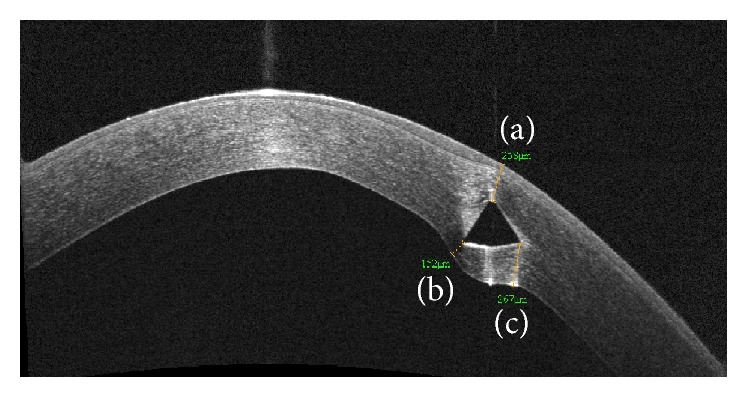
The segments traced to measure the distance from the apex to the anterior corneal surface (a) and the distance from the inner (b) and the outer (c) basal corners to the posterior corneal surface.

The HD line was displayed on the instrument monitor and the “distance” tool of the instrument software was selected. The mouse cursor was positioned at the Keraring apex, and a segment was traced to the anterior corneal surface. Using the mouse cursor, the distal point of segment was slowly dragged along the anterior corneal surface to identify the shortest segment. The length of this segment was recorded as the distance from the apex to the anterior corneal surface. The same procedure was done at the inner corner and the outer corner to measure the distance between them and the posterior corneal surface (Figure 3).

These measurements were taken by a second investigator (C.L.) in a separate session. The two sets of measurements were compared to assess interexaminer reproducibility. The first set of measurements was used to assess the difference with respect to the intended distance. The intended distance from the apex to the anterior corneal surface was the depth of the incision made to create the tunnel. The intended distance from the inner and the outer corners to the posterior corneal surface was the difference between the preoperative corneal thickness and the depth of the incision made to create the tunnel. For this calculation, the corneal thickness was measured on the preoperative pachymetry map. On this map, using the enface OCT image of the segment as a reference (Figure 2), the corneal thickness was measured at the point where the distance from the Keraring inner and outer corner to the posterior corneal surface was calculated.

### 2.3. Statistical Analysis

Statistical analysis was performed using STATA software package version 8.0 (StataCorp LP, College Station, TX, USA). The significance of differences between preoperative and postoperative data was assessed using Student's paired *t*-test for continuous variables and the Wilcoxon test for noncontinuous variables. The 95% limits of agreement (LoA) were calculated to estimate the agreement between examiners in measuring Keraring distance from the anterior and the posterior corneal surface and the agreement between the measured and the intended distance. The 95% LoA was calculated as described by Bland and Altman (95% LoA = mean difference ± 1.96 × SD) [14]. The absolute difference was calculated to assess the absolute value of the difference between the measured and the intended distance. A *χ*^2^ or Fisher's exact test was used to determine the differences in the proportion of eyes in which the measured distance fell within ±25 *μ*m and ±50 *μ*m of the intended distance. Differences were considered statistically significant when *p* value was less than 0.05.

## 3. Results

Overall, 26 Keraring ICRS were implanted: one per eye in 22 cases and 2 per eye in 2 cases. Surgery was uneventful and no major postoperative complications, such as corneal infections or ICRS extrusion, were observed.

The Keraring characteristics, UDVA, CDVA, and Km, of each case are shown in Table 1.

The Keraring SI-5 model (inner diameter 4.40 mm, base width 0.60 mm) was used in 21 cases and the SI-6 model (inner diameter 5.40, base width 0.80 mm) in 5 cases. The mean UDVA and CDVA were significantly improved (*p* < 0.001) after surgery. On average, UDVA improved by 0.19 ± 0.17 and CDVA by 0.22 ± 0.15. The postoperative mean Km was significantly reduced (*p* < 0.001) as compared with the preoperative value.

**Figure 4:**
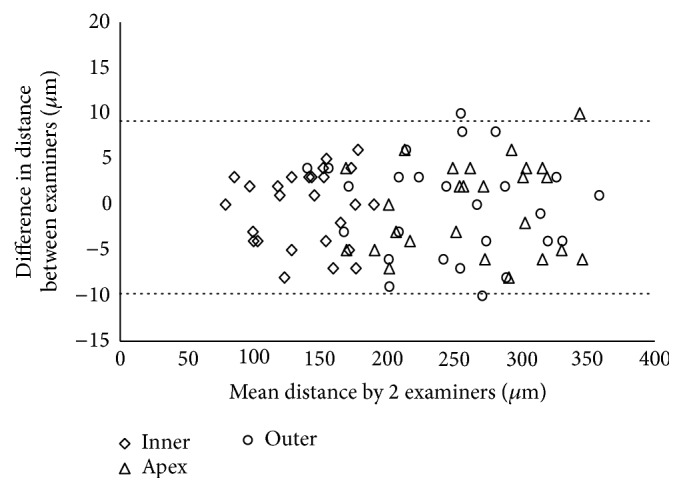
Scatterplot showing the differences between examiners' measurements of the distance from apex to anterior corneal surface (∆), from inner corner to posterior corneal surface (◊), and from outer corner to posterior corneal surface (○). Individual differences between examiners are plotted against the mean value obtained by both examiners together. The 95% LoA interval is represented with dotted lines.

The distance from Keraring to the anterior and the posterior corneal surface measured by the two examiners was not statistically different (*p* < 0.05). Individual differences between examiners are presented graphically through Bland-Altman plots (Figure 4).

The 95% LoA between examiners were −9.95 to 9.49 for the distance from the apex to the anterior corneal surface, −8.36 to 7.67 *μ*m for the distance from the inner corner to the posterior corneal surface, and −11.19 to 10.50 *μ*m for the distance from the outer corner to the posterior corneal surface.

The measured distance, intended distance, and absolute difference between the measured and the intended distance in each case are reported in Table 2.

The distance from the inner corner to the posterior corneal surface was not statistically different from the intended distance (*p* = 0.83). The distance from the apex to the anterior corneal surface was significantly less (*p* < 0.001) and the distance from the outer corner to the posterior corneal surface was significantly greater (*p* < 0.001) than the intended distance.

The mean absolute difference with respect to the intended distance was significantly lower (*p* < 0.001) for the distance from the inner corner to the posterior corneal surface as compared with those obtained for the other measurements. The measurements taken at the inner corner were within ±25 *μ*m and ±50 *μ*m of the intended distance in 15/26 (57.7%) and 24/26 (92.3%) cases, respectively. For the measurements taken at the apex and the outer corner, the proportions were significantly (*p* < 0.001) lower (3/26 (11.5%) and 5/26 (19.2%) cases, resp.).

**Figure 5:**
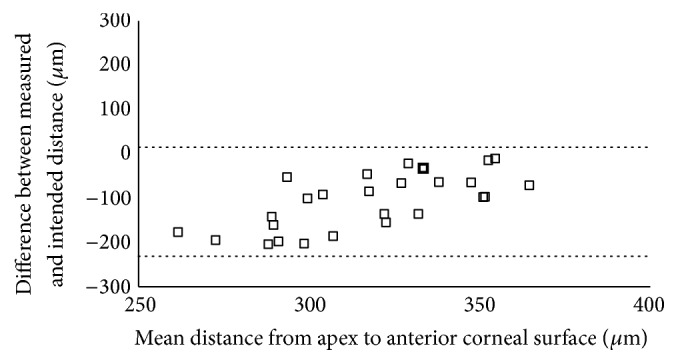
Scatterplot showing the differences between the measured and the intended distance from the Keraring apex to the anterior corneal surface. Individual differences between the measured and the intended distance are plotted against the mean value of the measured and the intended distance. The 95% LoA interval is represented with dotted lines.

**Figure 6:**
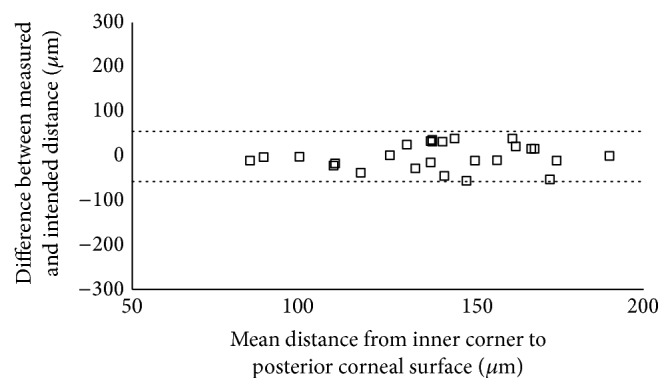
Scatterplot showing the differences between the measured and the intended distance from the Keraring inner corner to the posterior corneal surface. Individual differences between the measured and the intended distance are plotted against the mean value of the measured and the intended distance. The 95% LoA interval is represented with dotted lines.

**Figure 7:**
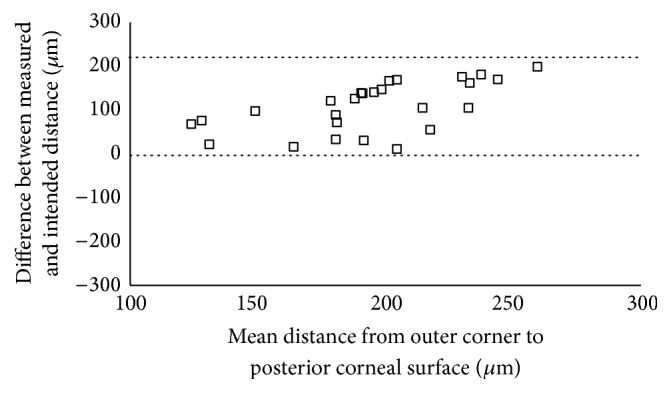
Scatterplot showing the differences between the measured and the intended distance from the Keraring outer corner to the posterior corneal surface. Individual differences between the measured and the intended distance are plotted against the mean value of the measured and the intended distance. The 95% LoA interval is represented with dotted lines.

The 95% LoA between the measured and the intended distance was −231.85 to 14.01 *μ*m for the distance from the apex to the anterior corneal surface, −57.61 to 55.15 *μ*m for the distance from the inner corner to the posterior corneal surface, and −3.35 to 220.04 *μ*m for the distance from the outer corner to the posterior corneal surface (Figures 5–7).

**Figure 8:**
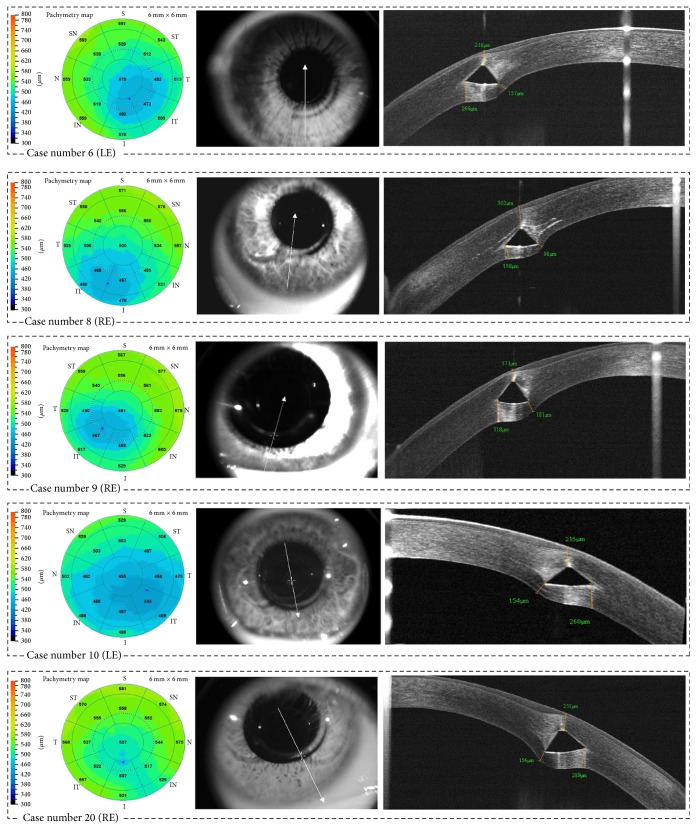
The pachymetry map and the Keraring OCT images (enface and high definition line) of cases # 6, # 8–10, and # 20. LE = left eye; RE = right eye.

The pachymetry map and the Keraring OCT images of 5 representative patients are shown in Figure 8.

## 4. Discussion

In the postoperative assessment of patients implanted with ICRS, it is essential to determine whether the segment is actually lying at the intended depth. Any error in implantation depth may reduce procedure efficacy and increase the risk of complications. Actual depth may differ from intended depth due to inaccurate measurement of corneal thickness preoperatively or to creation of the stromal tunnel at the wrong depth. Corneal thickness is conventionally measured using ultrasound pachymetry, which entails taking multiple measurements along the hypothetical tunnel area and can result in large interexaminer/intraexaminer variability in keratoconic corneas [15]. Tunnel depth may differ from that intended when a manual technique, or even femtosecond laser technology, is used for creating the tunnel [16] because technique accuracy may be reduced in the deep stroma [17–19].

In this study, the tunnel depth for Keraring implantation was planned using the pachymetry map created in RTVue 100. With a single and no-contact examination, it provides reliable pachymetric mapping over a corneal area 6 mm in diameter [13]. This area of analysis includes the tunnel area for implantation of the Keraring SI-5 and SI-6 models (inner diameter of 4.40 mm and 5.40 mm, resp.). Using this method, we were able to safely implant the ICRS in all patients.

After surgery, the measurements of Keraring distance from the anterior and the posterior corneal surface were highly reproducible. The interexaminer difference in measurements of the distance from the apex to the anterior surface and the distance from the inner and the outer corner to the posterior surface was small. This finding indicates that the method may be useful for assessing the ICRS position over time in reference to the anterior and the posterior cornea.

The distance of the inner corner from the posterior surface showed the best agreement with the intended distance (mean 138.85 ± 31.78 versus 140.08 ± 28.93 *μ*m; *p* = 0.83). The mean absolute difference with respect to the intended distance (23.54 ± 15.90 *μ*m) was significantly lower (*p* < 0.001) than that found for the other measurements and was within 25 *μ*m and 50 *μ*m in 57.7% and 92.3% of cases, respectively. The good agreement found for this measurement may be explained by the architecture and biomechanical properties of the posterior cornea [20, 21]. The posterior cornea has a low tensile strength and opposes low resistance to the pushing effect of the segment [22]. As a consequence, the posterior lamellae are slightly compressed behind the Keraring base, and the distance from the inner corner to the posterior surface is close to the intended distance.

The distance from the Keraring outer corner to the posterior corneal surface showed poor agreement with the intended distance. It was greater than intended, on average, by 108.35 *μ*m (*p* < 0.001). As compared with the inner corner, the outer corner was farther from the posterior corneal surface, on average, by 109.58 *μ*m. Using an IntraLase femtosecond laser, Gorgun et al. obtained similar results in 17 keratoconic eyes [9]. In their study, the outer corner was farther from the posterior surface, on average, by 88.0–111.7 *μ*m as compared with the inner corner. These findings show that the Keraring base is positioned obliquely with respect to the posterior corneal surface. It is unlikely that this position results from the creation of an oblique intrastromal tunnel. A tunnel created with the IntraLase should run parallel to the corneal surfaces because it is prepared while the cornea is applanate by the optical interface. The greater distance from the outer corner to the posterior corneal surface may be due to two factors. First, the outer corner might move forward during segment insertion into the tunnel or during the early postoperative period. Pérez-Merino et al. found that the Keraring base was tilted forward with respect to the iris plane, on average, by 6.8°  ±  2.6 at 7 days after surgery; small changes of tilt were observed between 7 and 90 days after surgery [11]. Second, the distance from the outer corner to the posterior surface might be overestimated due to the refractive distortion generated by the implant. The dewarp calculation of the instrument software uses a 1.337 refractive index for the cornea, but it does not take into account the fact that the implant refractive index is 1.487. As a consequence, the higher optical path difference through the implant increases the thickness in the peripheral areas of the corneas. Ortiz et al. reported that this refractive distortion may increase by up to 35 *μ*m the estimated corneal thickness [23]. In the OCT images, the refractive distortion generates a bulging of the posterior cornea. However, this artifact should be lower behind the inner corner as compared with the outer corner (Figure 8). The inner corner is closer to the central cornea, and the laser beam path through the implant is short when the distance from the inner corner to the posterior surface is evaluated. However, a distortion-corrected calculation is needed to determine the impact of this factor on the measurements taken behind the implant.

The distance from the apex to the anterior corneal surface is routinely measured to monitor the risk of complications such as ring migration and exposure. This measurement, however, agrees poorly with the intended distance. In the present study, the apex was closer to the anterior corneal surface than intended, on average, by more than 90 *μ*m. These findings are consistent with those reported previously [9, 10]. Probably, the apex depth is shallower than intended because the stromal lamellae above the Keraring are highly compressed. Compression of the anterior lamellae derives from the pushing effect of the apex and from the high rigidity of the most anterior part of the stroma that tends to maintain the anterior curvature and shape [20–24].

In 10 eyes operated for keratoconus, Pérez-Merino et al. measured the distance between the center of mass of the Keraring and the anterior corneal surface [11]. They found good agreement between measurements and incision depth; the absolute difference was 23.93 ± 23.49 *μ*m. This variability is comparable with the variability we noted for the distance from the inner corner to the posterior corneal surface. However, a comparative study is needed to establish the relationship between these measurements and to determine if they can be combined to verify whether the Keraring depth matches the intended depth in reference to the anterior and the posterior corneal surface.

The present study has several limitations. The Keraring depth was analyzed 3 months after surgery to minimize any effect of postoperative edema and inflammation on measurements. Nonetheless, the results might have been influenced by a slight change in implant depth in the first weeks after surgery. The tunnel was prepared using a manual technique in the majority of cases. The predictability of tunnel depth may be lower with the manual technique as compared with femtosecond laser technology. This factor might have inflated the differences between the measured and the intended distance. When we analyzed the 5 eyes operated on with femtosecond laser separately, the absolute difference with respect to the intended distance dropped to 19.40 ± 10.04 *μ*m for the measurements taken at the inner corner. Finally, the study did not investigate the agreement between the measured distance and the intended distance on the proximal and distal parts of the segment, where it might differ from that found on the central part. In any case, the difference should be small. Naftali and Jabaly-Habib found that segment depth does not change significantly on the proximal and distal parts as compared with the central part [10].

In conclusion, reproducible measurements of Keraring distance from the anterior and the posterior corneal surface were obtained with spectral-domain OCT. These measurements may be useful for assessing implant stability over time. The distance from the inner corner to the posterior corneal surface showed good agreement with the intended distance. This measurement may be used to determine whether, in reference to the posterior corneal surface, segment depth matches intended depth.

## Figures and Tables

**Table 1:** Keraring characteristics, uncorrected distance visual acuity (UDVA), corrected distance visual acuity (CDVA), and mean keratometry (Km) of each patient.

Case	Eye	Keraring		UDVA		CDVA		Km (D)	
		Model	Arc length (°)/ thickness (*μ*m)	Pre	Post	Pre	Post	Pre	Post
1	RE	SI-5	160/300	0.04	0.10	0.50	0.80	50.15	48.68
2	LE	SI-5	120/200	0.10	0.10	0.60	0.70	50.82	49.77
		SI-5	120/200						
3^*∗*^	LE	SI-5	160/250	0.10	0.10	0.40	0.70	49.31	45.12
4^*∗*^	LE	SI-6	150/150	0.10	0.20	0.70	1.00	43.89	43.48
5^*∗*^	RE	SI-5	160/300	0.30	0.80	0.50	1.00	45.27	43.41
6	LE	SI-5	160/250	0.10	0.20	0.60	1.00	42.98	41.19
7	RE	SI-5	160/250	0.30	0.60	0.70	0.90	51.49	49.00
8	RE	SI-5	160/250	0.08	0.60	0.60	0.90	45.39	43.68
	LE	SI-6	150/250	0.10	0.30	0.50	0.90	47.19	44.07
9	RE	SI-5	160/300	0.04	0.40	0.60	0.80	48.06	45.62
10	RE	SI-5	160/250	0.10	0.40	0.80	1.00	48.44	47.90
	LE	SI-5	160/200	0.20	0.40	0.90	1.00	49.45	46.15
11	RE	SI-5	160/300	0.10	0.10	0.70	0.80	50.10	49.31
12	RE	SI-6	150/200	0.40	0.80	0.90	0.90	46.48	45.74
	LE	SI-6	150/250	0.40	0.40	0.80	1.00	45.41	41.00
13	RE	SI-5	120/250	0.10	0.20	0.60	0.80	51.70	50.30
14	LE	SI-5	160/300	0.08	0.30	0.40	0.50	46.40	44.40
	LE	SI-5	90/150						
15	LE	SI-5	160/250	0.20	0.30	0.60	0.80	45.95	44.20
16^*∗*^	LE	SI-5	160/250	0.10	0.60	0.50	0.80	50.30	50.00
17^*∗*^	RE	SI-5	160/250	0.06	0.20	0.30	0.80	46.75	45.60
18	LE	SI-5	210/200	0.06	0.30	0.40	0.70	49.04	46.83
19	RE	SI-5	160/250	0.10	0.40	0.40	0.80	49.40	45.96
20^*∗*^	RE	SI-6	150/300	0.10	0.40	0.80	0.80	46.12	45.17
21	RE	SI-5	160/300	0.10	0.10	0.50	0.70	45.64	41.19

MEAN				0.14	0.35	0.60	0.84	47.74	45.74
SD				0.10	0.21	0.17	0.13	2.44	2.79
Range				0.04–0.4	0.1–0.80	0.3–0.9	0.5–1	42.98–51.7	41–50.3

RE: right eye. LE: left eye. ^*∗*^Eyes with intrastromal tunnel created using femtosecond laser. SD: Standard Deviation. D: diopters.

**Table 2:** Keraring measured and intended distance from the anterior and the posterior corneal surfaces.

Case	Eye	Intended distance (*μ*m)		Distance apex/ anterior surface (*μ*m)		Distance inner corner/ posterior surface (*μ*m)		Distance outer corner/ posterior surface (*μ*m)	
		Anterior surface	Posterior surface	Measured	Absolute difference	Measured	Absolute difference	Measured	Absolute difference
1	RE	391	136	205	186	98	38	225	89
2	LE	333	176	287	46	120	56	207	31
		335	199	313	22	146	53	210	11
3^*∗*^	LE	350	162	258	92	152	10	267	105
4^*∗*^	LE	370	147	306	64	119	28	328	181
5^*∗*^	RE	399	122	201	198	154	32	260	138
6	LE	377	125	216	161	157	32	266	141
7	RE	405	180	250	155	169	11	285	105
8	RE	400	100	302	98	98	2	198	98
	LE	358	118	343	15	101	17	239	121
9	RE	366	142	171	195	181	39	318	176
10	RE	344	118	167	177	143	25	285	167
	LE	357	121	215	142	154	33	260	139
11	RE	399	145	328	71	130	15	217	72
12	RE	400	152	303	117	173	21	314	162
	LE	401	160	198	203	176	16	359	199
13	RE	323	164	270	53	119	45	197	33
14	LE	356	125	255	101	126	1	251	126
	LE	354	190	321	33	190	0	245	55
15	LE	362	90	296	66	87	3	166	76
16^*∗*^	LE	360	156	349	11	145	11	172	16
17^*∗*^	RE	392	159	188	204	175	16	329	170
18	LE	383	120	318	65	98	22	142	22
19	RE	358	90	273	85	79	11	158	68
20^*∗*^	RE	387	120	251	136	156	36	289	169
21	RE	400	125	264	136	164	39	272	147

MEAN		371.54	140.08	263.38	108.92	138.85	23.54	248.42	108.35
SD		24.42	28.93	54.16	62.72	31.78	15.90	57.32	56.99
RANGE		323–405	90–199	167–349	11–204	79–190	0–56	142–359	11–199

RE: right eye. LE: left eye. ^*∗*^Eyes with intrastromal tunnel created using femtosecond laser. SD: Standard Deviation.
